# A rare case of primary unilateral conjunctival small lymphocytic lymphoma: A case report

**DOI:** 10.1016/j.ijscr.2024.110812

**Published:** 2024-12-30

**Authors:** Teketel Tadesse Geremew, Woldie Jember Zewdie, Abebe Melis Nisiro, Ghion Getenet Engida, Tigist Gutema Tesgera

**Affiliations:** a Department of Pathology, Hawassa University Comprehensive Specialized Hospital, Hawassa, Sidama, Ethiopia; b Department of Pathology, Worabe Comprehensive Specialized Hospital, Ethiopia; c Department of Internal Medicine, Hawassa University Comprehensive Specialized Hospital, Hawassa, Sidama, Ethiopia

**Keywords:** Ocular lymphoma, Small lymphocytic lymphoma, Conjunctiva, Low grade non-Hodgkin's lymphoma, Case report

## Abstract

**Introduction and importance:**

Orbital lymphomas, are extranodal lymphomas primarily involving the ocular adnexa, which includes conjunctiva, eyelids, eyelashes, eyebrows, lacrimal glands, retro-orbital soft tissues, and the extraocular muscles. Ocular adnexal lymphomas can be primary or secondary. This case is important because there are few case reports in the world, and it is among the first case to be reported from Ethiopia.

**Case presentation:**

Our case is a 32-year-old female patient from the Ethiopia who has presented with painless slowly growing conjuctival mass over the left eye. Histopathologic and IHC examination confirm the diagnosis of primary conjuctival small lymphocytic lymphoma (SLL).

**Clinical discussion:**

The majority of Ocular adnexal lymphomas are unilateral, with bilateral lesions occurring in about 7 %–24 % of all cases. Ocular adnexal lymphomas are mostly low-grade B-cell non-Hodgkin lymphomas, with approximately half being extranodal marginal zone B-cell lymphomas, with small lymphocytic lymphomas being much less common. The treatment options include radiotherapy, immunotherapy, chemotherapy, or a combination of these treatments. The gold standard treatment of isolated conjunctival lymphoma after resection is localized external-beam radiation therapy.

**Conclusion:**

SLLs of the ocular adnexa are rare, especially in the conjunctiva, with few cases reported in the published literature. The treating physician's should consider primary lymphomas are a possible cause of salmon colored mass in the conjunctiva specially those having history of chronic conjunctivitis. Histopathology with immunohistochemistry confirms the diagnosis of ocular lymphoma.

## Highlights

• Ocular adnexal lymphomas (OAL) account for 2% of all non-Hodgkin lymphomas, and about 6-8% of extranodal lymphomas
• Orbital lymphomas, are extranodal lymphomas primarily involving the ocular adnexa.
• Ocular adnexal lymphomas can be primary or secondary.
• The most common type of ocular adnexal lymphomas is the extranodal marginal zone B-cell lymphoma but small lymphocytic lymphoma is rare

## 1 Introduction

Ocular adnexal lymphomas (OALs), also called orbital lymphomas, are extranodal lymphomas primarily involving the ocular adnexa, which includes conjunctiva, the contents of the orbit, eyelids, eyelashes, eyebrows, lacrimal glands, retro-orbital soft tissues, and the extraocular muscles [1,2]. OALs can be primary or secondary. They are considered primary if the involvement of lymphoma is confined to the ocular adnexa alone and secondary if there is lymphoma of the identical type present at another site [1].

Lymphomas are of two main types, Hodgkin and non-Hodgkin lymphoma. Non-Hodgkin lymphomas are more common and approximately one-third of all non-Hodgkin lymphoma cases are extranodal [3]. In the ocular adnexa, the conjunctiva is an important site of extranodal lymphoma development, and it accounts for approximately 25 % of the OALs [4]. The most common type of OAL is the extranodal marginal zone B-cell lymphoma (EMZL), also known as mucosa-associated lymphoid tissue lymphoma, followed by follicular lymphoma, mantle cell lymphoma, and diffuse large B-cell lymphoma, each accounting for approximately 10 % of primary OALs [5].

Small lymphocytic lymphomas (SLLs) of the ocular adnexa are rare, with few cases reported in the published literature [1,6]. To our knowledge, we report the first case of the primary unilateral conjunctival SLL in Ethiopia.

This case report is written following the SCARE criteria [7].

## 2 Case presentation

Our case is a 32-year-old female patient from Ethiopia who has presented with painless slowly growing conjuctival mass over the left eye. The patient experienced intermittent left eye discomfort for two years. Our patient had childhood history of repetitive treatment for keratoconjuctivitis. Otherwise, the patient didn't have any history of trauma to the site. She doesn't have any history of diabetes mellitus, hypertension, or immunocompromise state.

### 2.1 Physical findings

Vital signs were all within the normal limits.

**Fig. 1:**
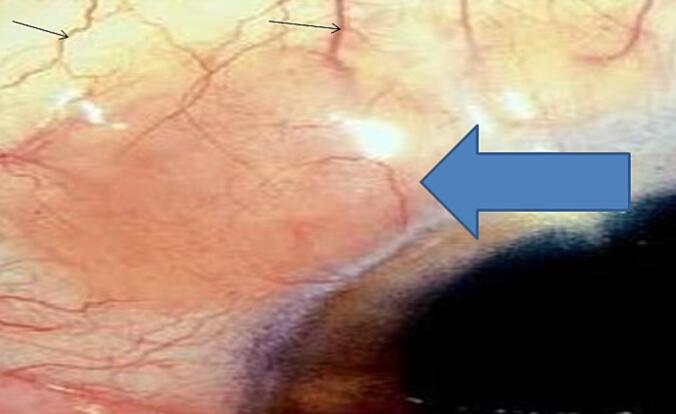
Gross image shows salmon colored mass (blue arrow) surrounded by dilated feeder vessels (black arrow).

Eye examination: painless salmon-colored left conjunctival lesion surrounded by dilated vessels which appeared to be feeder vessels (Fig. 1). The visual acuity was within normal limits and fundal examination is unremarkable.

### 2.2 Laboratory investigations were done on investigation

Full blood count (WBC-8.6 × 10 9/L, Hgb-12.5 g/dl, platelet- 265 × 10 9/L), liver and renal function tests were within the normal range. Antibody tests for HIV and VDRL infections were negative.

For this, the patient underwent a complete surgical excisional biopsy using the “no touch” technique with 3-mm conjunctival margin, followed by cryotherapy of the conjunctival margins. The “no touch” technique was employed to avoid seeding of any possible tumor, whereby only the surrounding normal tissue was held with forceps, and the lesion was not touched. The excisional biopsy of the lesion was followed by the application of absolute alcohol using a cotton-tipped applicator for 60 s. A balanced salt solution was not employed during surgery to avoid liquid dispersion of any possible tumor cells.

**Fig. 2:**
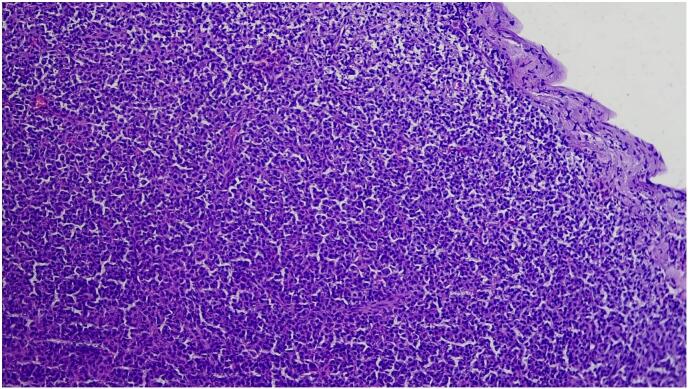
Low power view (4×) shows diffuse sheets of monotonous small lymphoid cells.

**Fig. 3:**
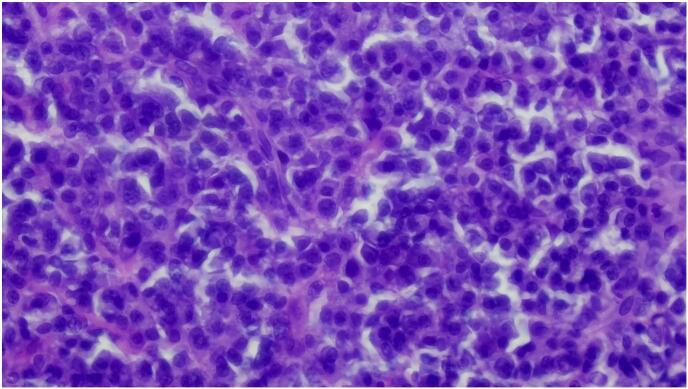
High power view (40×) shows diffuse sheets of small mature lymphocytes with round nucleus, clumped chromatin, only small nucleoli and scant cytoplasm, intermixed with slightly larger cells with more dispersed chromatin and small to prominent nucleoli.

**Fig. 4:**
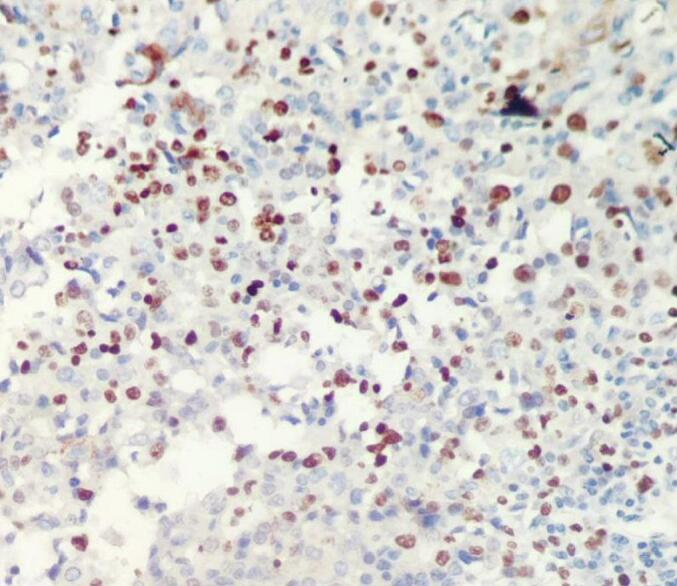
High power view (40×) shows Ki 67 nuclear stain.

The lesional tissue was fixed in 10 % neutral-buffered formalin, serial sectioned done, and stained with hematoxylin and eosin, and the pathology revealed diffuse sheets of small, mature lymphocytes with round nucleus, clumped chromatin, only small nucleoli and scant cytoplasm, intermixed with slightly larger cells with more dispersed chromatin and small to prominent nucleoli (Fig. 2, Fig. 3). On immunohistochemical analysis, the lesional cells were immunopositive for CD5, BCL2, CD23, CD5, and PAX-5, and immunonegative for BCL 6, CD10 and cyclin D1. The Ki-67 proliferation index was up to 20–30 % (Fig. 4). The final histopathological diagnosis of conjunctival SLL was made. Due to the lack of evidence of any systemic involvement, no bone marrow aspiration/biopsy was done. A final clinical diagnosis of primary unilateral conjunctival SLL was made. Localized external-beam radiation therapy was given for our patient. The patient is currently on a regular follow-up with smooth post-op condition.

## 3 Discussion

Ocular adnexal lymphomas (OAL) account for 2 % of all non-Hodgkin lymphomas, and about 6–8 % of extranodal lymphomas [8]. The primary involvement of the conjunctiva by lymphoma, comprises about one third of ocular adnexal lymphoma, and 1.5 % of all conjunctival tumors [9]. Approximately 73 % of OALs arise as a primary ocular adnexal disease, whereas 27 % are the result of metastatic spread [2].

The majority of OALs are unilateral, with bilateral lesions occurring in about 7 %–24 % of all cases [10]. Ocular adnexal lymphomas are mostly low-grade B-cell non-Hodgkin lymphomas [5]. The three most common primary conjuctival non-Hodgkin's lymphoma types are extranodal marginal zone lymphoma accounting for 60 % of the cases, follicular lymphoma which accounts for 10 % of cases, diffuse large B-cell lymphoma accounting for 9 %, with small lymphocytic lymphomas being much less common and exact incidence is not well known due to the rarity of the case [5] and our case is among this extremely rare case of primary conjuctival small lymphocytic lymphomas. One of the proposed risk factors for conjuctival lymphoma is autoimmune disease, other include chlamydia psittaci, helicobacter pylori, and HIV infection. The development of conjunctival lymphomas is frequently associated with chronic inflammation secondary to either endogenous or exogenous antigens [4]. They characteristically present as a chronic, painless, sessile, flesh-colored “salmon-patch” subepithelial conjunctival mass lesion [11]. The diagnosis of conjunctival lymphoma is sometimes clinically difficult, as reactive lymphoid hyperplasia and lymphoma share similar features, making tissue biopsy and histopathology examination critical for diagnosis [4]. However, features such as feeder vessels and rapid growth seen in conjunctival reactive lymphoid hyperplasia are not typically seen in conjunctival lymphomas [12]. Our patient had a painless, slowly growing, salmon-colored conjunctival peribulbar lesion surrounded by dilated vessels which appeared to be feeder vessels; therefore, the initial clinical suspicion was of an inflammatory process. The diagnosis of conjunctival SLL was thereby made on histopathological examination.

Once the diagnosis of conjunctival lymphoma has been confirmed, a complete workup by an oncologist is recommended to determine the existence and extent of any systemic disease. A complete clinical workup should include a CBC, serum chemistry studies (including lactate dehydrogenase), and computed tomography, and full-body PET scan [12]. Our patient, on further evaluation, revealed no systemic involvement and all her blood workup was within normal limits. Therefore, his final clinical diagnosis was primary unilateral conjunctival SLL.

The treatment options for OALs include radiotherapy, immunotherapy, chemotherapy, or a combination of these treatments. The gold standard treatment of isolated conjunctival lymphoma after resection is localized external-beam radiation therapy [4]. Occasionally, an excisional biopsy and periodic observation might suffice for a unilateral isolated conjunctival lymphoma if the conjunctival lesion is relatively small and circumscribed, and clear excisional margins have been achieved. However, this approach is associated with a high risk of recurrence and progression of the conjunctival lymphoma along with the development of systemic lymphoma in the future without adjuvant treatment due to subclinical invasion of tumor into the surrounding tissues [13,14] There have been rare case reports of spontaneous regression of conjunctival lymphoma following excisional biopsy [14].

The 5-year survival rate for OAL varies between 50 % and 94 %, depending on the patient's age, histologic subtype of the lymphoma, tumor, node, metastasis stage at diagnosis, and type of treatment given [7]. Conjunctival lymphomas have an excellent prognosis, with a 90 % recurrence-free rate in some studies after a year of follow-up [15]. The most important prognostic factor in conjunctival lymphomas is the histological subtype of the lesion, with primary localized conjunctival low-grade EMZL and follicular lymphomas having the best outcome after treatment [15,16]. Studies evaluating the treatment and prognosis of conjunctival SLL have not yet been published due to the rarity of this lesion.

## 4 Conclusions

SLLs of the ocular adnexa are rare, especially in the conjunctiva, with few cases reported in the published literature. Conjuctival lymphomas may be clinically missed due to a lack of specific symptoms. Therefore, ophthalmologists should keep a low clinical threshold for suspecting lymphoma in patients with chronic conjunctival lesions such as chronic conjunctivitis. Tissue biopsy and histopathology examination are critical for diagnosis. Staging and systemic evaluation are important for formulating a proper treatment plan. Moreover, finally, long-term follow-up is necessary for conjunctival lymphomas, as systemic lymphoma may develop after many years.

## Abbreviations

OALs: Ocular adnexal lymphomas
SLL: Small lymphocytic lymphoma
HIV: Human immunodeficiency virus
VDRL: Venereal Disease Research Laboratory test

## CRediT authorship contribution statement

Teketel Tadesse Geremew, MD - Study concept and design, writing the paper, literature review and editing and critical review of the paper
Wolde Jember Zewdie and Abebe Melis Nesiro, MD - Involved in acquisition of data, literature review of the paper, writing and drafting the paper, editing and critical review of the paper
Tigist Gutema Tesgera and Ghion Getenet Engida, MD - Literature review of the paper, writing and drafting the paper, editing and critical review of the paper.

## Patient consent

The report was done with patient's consent.

## Ethical approval

No ethics approval was needed as the case was encountered incidentally and it doesn't involve any human or animal experiment.

## Guarantor

Teketel Tadesse Geremew, MD.

## Funding

None.

## Declaration of competing interest

The authors declare that there are no conflicts of interest on this case report.
